# Towards “Green” ANFO: Study of Perchlorates and Inorganic Peroxides as Potential Additives

**DOI:** 10.3390/molecules28155636

**Published:** 2023-07-25

**Authors:** Magdalena Fabin, Paweł Skóra, Mateusz Polis, Roman Zakusylo, Agnieszka Stolarczyk, Tomasz Jarosz

**Affiliations:** 1 Department of Physical Chemistry and Technology of Polymers, Silesian University of Technology, 44-100 Gliwice, Polandagnieszka.stolarczyk@polsl.pl (A.S.); 2 Department of Inorganic Chemistry, Analytical Chemistry and Electrochemistry, Silesian University of Technology, 44-100 Gliwice, Poland; 3 Explosive Techniques Research Group, Łukasiewicz Research Network-Institute of Industrial Organic Chemistry, 42-693 Krupski Młyn, Poland; 4 Shostka Institute, Sumy State University, 41100 Shostka, Ukraine; r.zakusylo@ishostka.sumdu.edu.ua

**Keywords:** ANFO, additive, shock sensitivity, perchlorate, peroxide

## Abstract

Ammonium nitrate–fuel oil (ANFO) explosives are inexpensive and readily produced, but are highly prone to misfires, with the remaining explosive being a significant risk and environmental contaminant. In this work, studies on various additives, such as selected perchlorates and inorganic peroxides, which are intended to lower the susceptibility of ANFO to misfires by increasing its sensitivity to shock, have been conducted. These studies showed the viability of using these additives in ANFO, allowing for conducting shock wave sensitivity tests for bulk charges in the future. We investigated the effects of introducing these additives into ANFO (on its sensitivity), as well as thermal and energetic properties. We observed minor increases in friction and impact sensitivity, as well as a moderate reduction in the decomposition temperature of the additive-supplemented ANFO in comparison to unmodified ANFO.

## 1. Introduction

Ammonium nitrate–fuel oil (ANFO) explosives are widely used in blasting operations, particularly those conducted for the purpose of open-pit and underground mining [1,2]. This popularity stems from the low unit costs of ANFO components and the simplicity of producing ANFO. An added benefit of employing ANFO as a blasting agent is that problematic waste products, such as spent oils, may be used as fuels for ANFO, without having a significant adverse effect on the performance of this type of explosive [3,4]. Moreover, raw materials of plant origins, such as wood dust, wheat flour, or rape seeds, can be used as fuels [5].

Despite the above advantages, ANFO is not free from drawbacks, as it is highly susceptible to water [6], exhibits relatively low performance (typical velocity of detonation is on the order of 2500 m/s) [7], and is prone to misfires [8]. Misfires result in unreacted or partially decomposed explosive that remains in boreholes, constituting a significant risk [9]. This remnant explosive may either smoulder and spontaneously undergo detonation at an unknown time interval or may be accidentally initiated during further activity (e.g., drilling boreholes, blasting operations, disposal of spoil) at the blasting site [10]. The remnants of ANFO left after a misfire are a significant issue in terms of environmental contamination [8], even if that is a more indirect and long-term risk.

In order to ameliorate the drawbacks of ANFO, significant research efforts have been (and are continuously being) undertaken, as seen in our earlier work, dedicated to reviewing the recent literature on the subject. Significant research efforts have been conducted on investigating the supplementation of ANFO with a variety of additives, such as chlorides and sulphates [11,12], and a measure of success was achieved in controlling the thermal stability of such modified ANFO. However, information about the impact of those additives on the explosive parameters of ANFO is relatively scarce. Most notably, among the strategies proposed to improve the energetic parameters of ANFO, reports on its ability to sustain detonation (expressed as, e.g., its critical diameter or shock sensitivity) are particularly rare [5].

An important gap in the state of the art is that the use of perchlorates as additives to ANFO has not been investigated in much detail. Moreover, no works are available on the use of stable inorganic peroxides, such as sodium carbonate peroxyhydrate (SPC) or sodium perborate (SPB) for this purpose, even though the two compounds are relatively common products of the chemical industry [13]. These compounds are of significant interest due to their facile decomposition, which results in the formation of significant amounts of gaseous products. The incipient microbubbles of evolving gases can act as hot-spots, improving the susceptibility of the modified ANFO to shock.

Consequently, the exploration of such substances as additives to ANFO is expected to be of significant importance in the development of misfire-resistant ANFO. In this work, we present the results of our initial investigation of the influence of four readily-decomposing additives (Mg(ClO_4_)_2_, Ba(ClO_4_)_2_, SPB, and SPC) on the most fundamental properties of ANFO, produced using two types of organic fuels (liquid paraffin and naphtha).

## 2. Results and Discussion

### 2.1. Scanning Electron Microscopy

**Figure 1:**
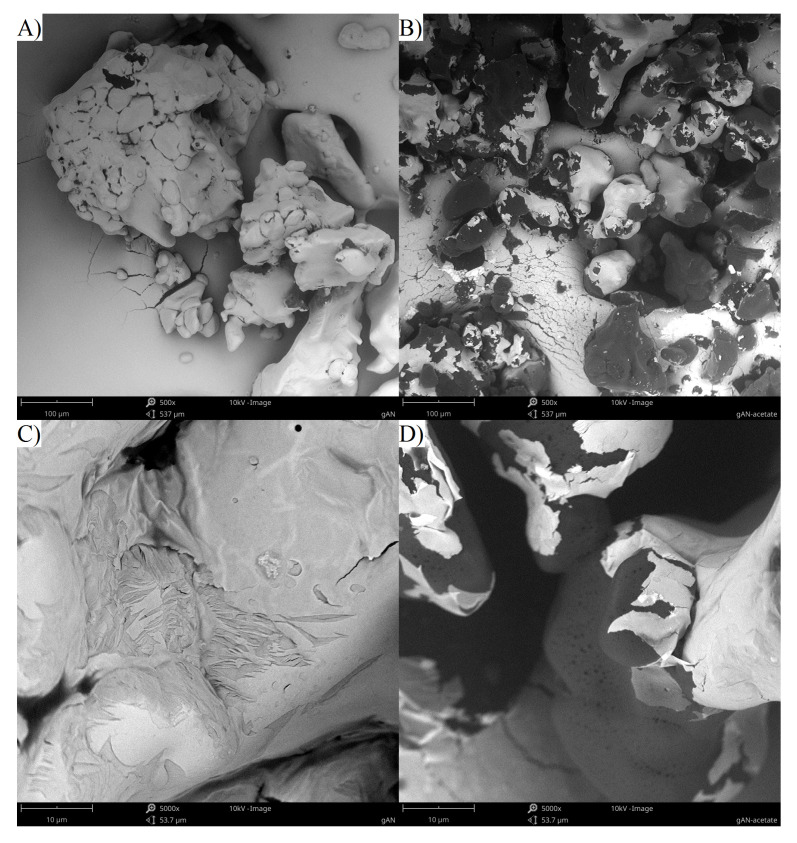
Compilation of SEM images for gAN (**A**,**C**) and gAN treated with acetone (**B**,**D**), as in the case of the procedure used for introducing additives. Magnifications of 500× (**A**,**B**) and 5000× (**C**,**D**) for comparison.

The ground ammonium nitrate (gAN) fraction has the form of non-porous, rough grains, resulting from the mechanical grinding of the AN prills (Figure 1A,C). Treatment with acetone results in the gAN particles becoming densely riddled with small (d < 1 μm) pores (Figure 1D). This treatment also resulted in trace amounts of acetone becoming entrapped in the gAN fraction, despite subsequent drying steps. This entrapped acetone evaporates under the high vacuum of the SEM chamber, resulting in the gold layer that was deposited on the sample surface being torn, revealing the non-conductive surfaces of the gAN particles (dark parts of the surface of the particles).

**Figure 2:**
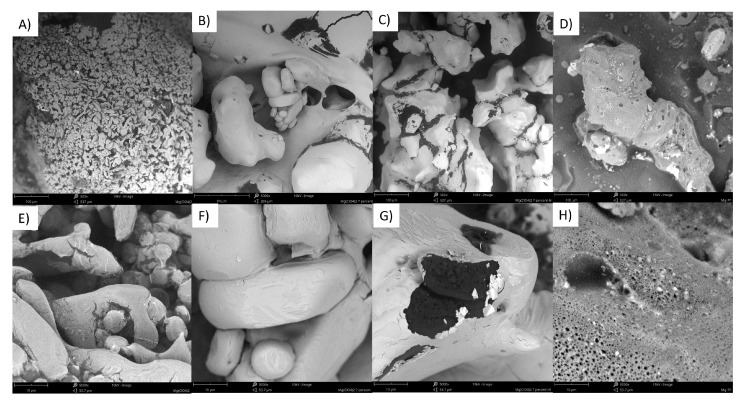
Compilation of SEM images for raw Mg(ClO_4_)_2_ (**A**,**E**), gAN supplemented with 7 wt. % Mg(ClO_4_)_2_ (**B**,**F**) and ANFO produced from gAN supplemented with 7 wt. % Mg(ClO_4_)_2_ using naphtha (**C**,**G**) and liquid paraffin (**D**,**H**) as fuel. Magnifications of 500× (**A**–**D**) and 5000× (**E**–**H**) for comparison.

The raw magnesium perchlorate (Figure 2A,E) has a granular morphology, likely due to the process used to achieve grain sizes within the micrometric range. Once it is applied as an additive, it appears to precipitate on the surface of the gAN particles (Figure 2B,F), producing grains with apparently non-porous surfaces. The introduction of either fuel, i.e., naphtha (Figure 2C,G) or liquid paraffin (Figure 2D,H) resulted in at least partial dissolution of the Mg(ClO_4_)_2_ coating on the gAN surface, restoring the porous microstructure. The effect of coating gAN pores and the re-opening of the pores after the introduction of fuel is also observed in samples supplemented with 1 wt. % (Figure A3) and 5 wt. % (Figure A4) of this additive. The porosity of the gAN particles after the introduction of either fuel appears to decrease on the order of 1 wt. % > 5 wt. % > 7 wt. %, which is consistent with the gradual saturation of the fuel with the dissolved Mg(ClO_4_)_2_.

In the case of barium perchlorate (Figure A5 and Figure A6), the raw additive is in the form of larger, highly porous granules. Upon its addition to gAN, the gAN particle surfaces do not lose the porosity that originates from treatment with acetone, as observed in the case of magnesium perchlorate. Instead, small particles of Ba(ClO_4_)_2_ appear to be deposited on the surface of the gAN particles.

The SPB-supplemented samples (Figure A7, Figure A8 and Figure A9) show the lowest porosity of all compositions, with clear pores appearing only in paraffin-containing systems. In the case of SPC samples (Figure A10, Figure A11 and Figure A12), the addition of both paraffin and naphtha improves the porosity of the systems.

### 2.2. DTA-TG

**Figure 3:**
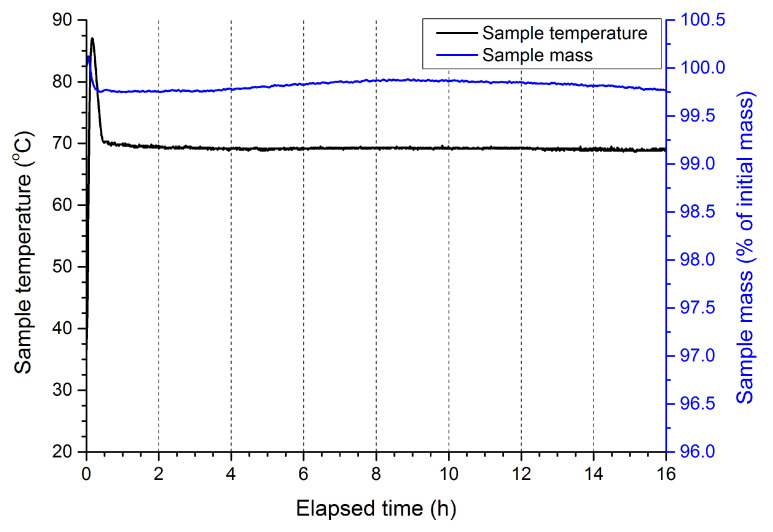
Thermogram recorded for ANFO produced using liquid paraffin as fuel.

**Figure 4:**
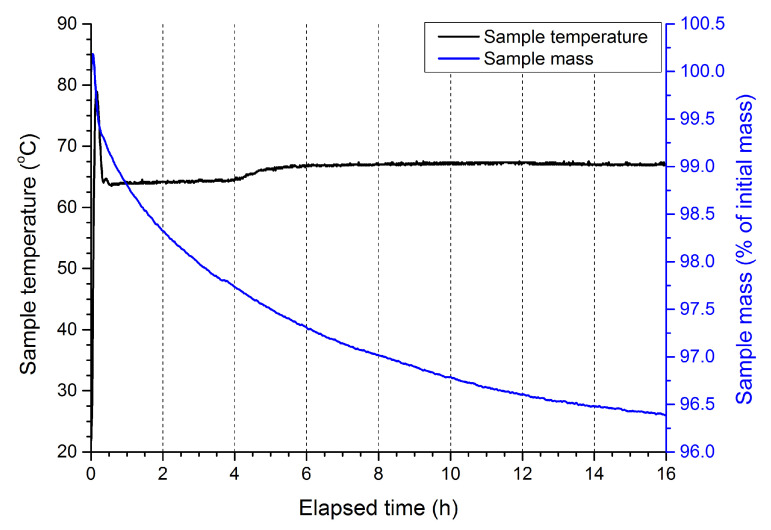
Thermogram recorded for ANFO produced using naphtha as fuel.

In all cases, an initial temperature spike of approx. 10–15 K magnitude is observed at the beginning of the measurement (Figure 3, Figure 4, Figure A1 and Figure A2). This is caused by the inertia of the utilised instrument, which was heated from room temperature up to 65–70 °C at a rate of 10 K/min. Depending on the investigated sample, various changes to the mass of the sample are observed over the course of the measurement. The magnitude of those changes does not exceed 0.25% and 4% of the initial sample mass, respectively, for samples utilising liquid paraffin and naphtha. In the case of ANFO produced using liquid paraffin, the mass loss is likely related to the evaporation of water that was adsorbed on the sample surface when the samples were transferred from the desiccator to the DTA-TG vessel. In the case of ANFO produced using naphtha, the larger observed mass loss is due to the lowest boiling fractions being boiled off, as expected, based on the boiling point of the naphtha specified by the producer (<90 °C).

Comparison of the recorded DTA-TG data for samples supplemented with additives reveals that the inclusion of the selected additives into the gAN fraction of the ANFO does not lead to any increased sample mass loss compared to the mass loss seen for non-supplemented gAN-based ANFO. In the case of samples containing 7 wt. % of SPC, even though no appreciable sample mass loss was observed, the sample was found to change its colour. Consequently, the use of SPC at this level of concentration was omitted from further investigations. The other SPC-supplemented samples (containing 1 wt. % and 5 wt. % SPC) did not exhibit such behavior, even upon prolonged heating.

### 2.3. Determination of Sample Decomposition Temperature and Pseudo-Activation Energy

**Figure 5:**
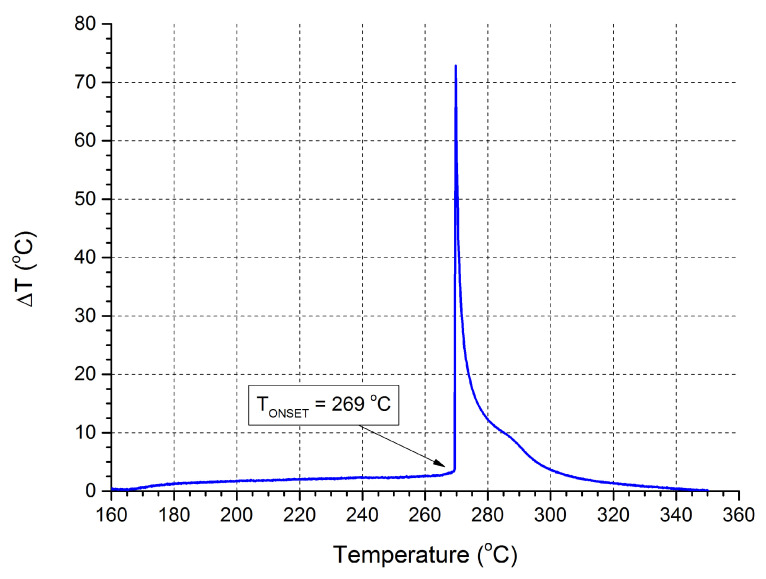
Thermogram recorded during the investigation of the ignition/explosion temperature of an ANFO sample produced using liquid paraffin as fuel and supplemented with 7 wt. % of Mg(ClO_4_)_2_ with a heating rate of 10/min). ΔT represents the difference between the temperature measured directly above the surface of the sample and the temperature of the heating block.

**Figure A1:**
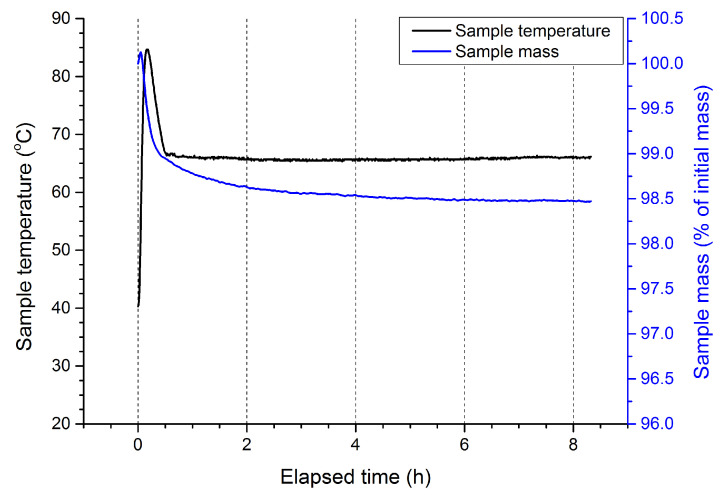
Thermogram recorded for ANFO produced using liquid paraffin as fuel and supplemented with 7 wt. % of Ba(ClO_4_)_2_.

**Figure A2:**
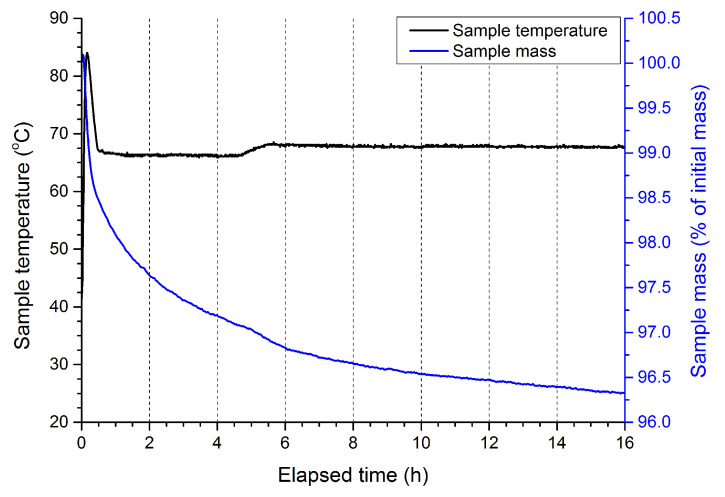
Thermogram recorded for ANFO produced using naphtha as fuel and supplemented with 7 wt. % of Ba(ClO_4_)_2_.

**Figure A3:**
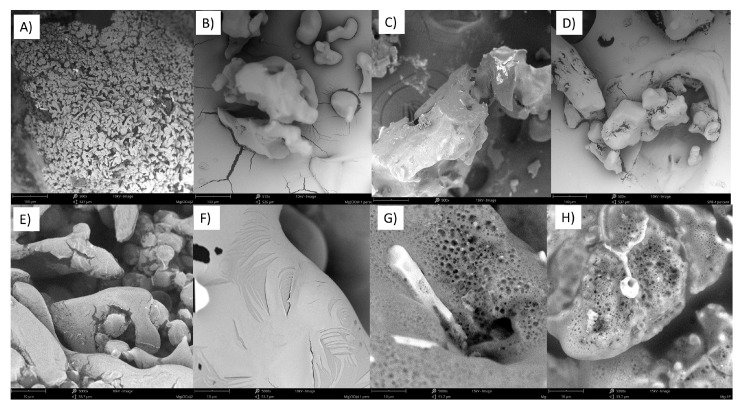
Compilation of SEM images for raw Mg(ClO_4_)_2_ (**A**,**E**), gAN supplemented with 1 wt. % Mg(ClO_4_)_2_ (**B**,**F**), and ANFO produced from gAN supplemented with 1 wt. % Mg(ClO_4_)_2_ using naphtha (**C**,**G**) and liquid paraffin (**D**,**H**) as fuel. Magnifications of 500× (**A**–**D**) and 5000× (**E**–**H**) for comparison.

**Figure A4:**
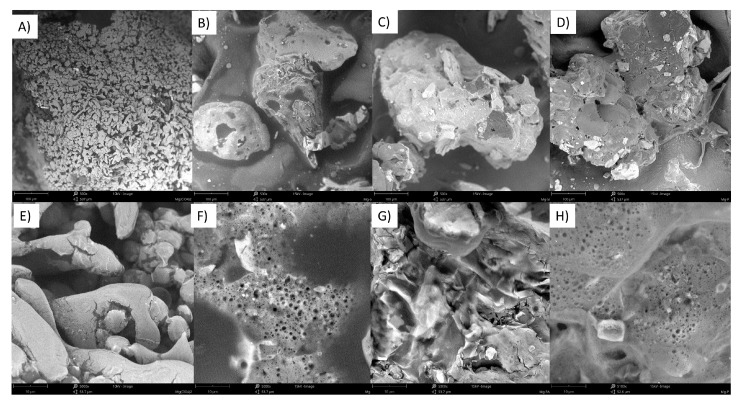
Compilation of SEM images for raw Mg(ClO_4_)_2_ (**A**,**E**), gAN supplemented with 5 wt. % Mg(ClO_4_)_2_ (**B**,**F**) and ANFO produced from gAN supplemented with 5 wt. % Mg(ClO_4_)_2_ using naphtha (**C**,**G**) and liquid paraffin (**D**,**H**) as fuel. Magnifications of 500× (**A**–**D**) and 5000× (**E**–**H**) for comparison.

**Figure A5:**
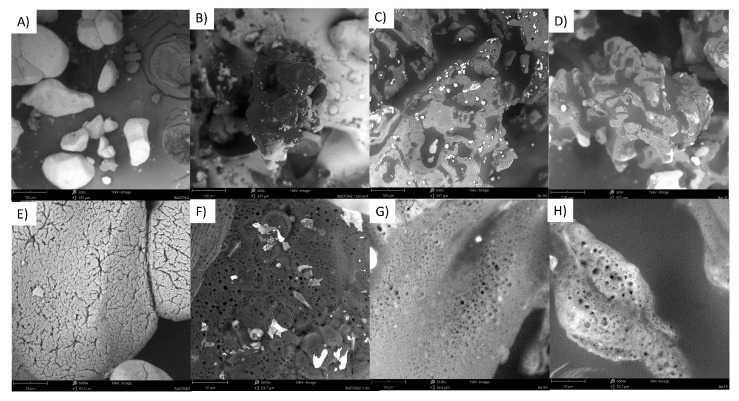
Compilation of SEM images for raw Ba(ClO_4_)_2_ (**A**,**E**), gAN supplemented with 1 wt. % Ba(ClO_4_)_2_ (**B**,**F**) and ANFO produced from gAN supplemented with 1 wt. % Ba(ClO_4_)_2_ using naphtha (**C**,**G**) and liquid paraffin (**D**,**H**) as fuel. Magnifications of 500× (**A**–**D**) and 5000× (**E**–**H**) for comparison.

**Figure A6:**
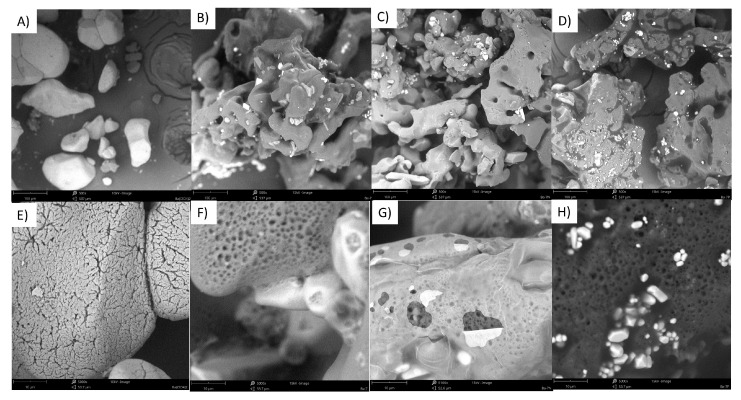
Compilation of SEM images for raw Ba(ClO_4_)_2_ (**A**,**E**), gAN supplemented with 7 wt. % Ba(ClO_4_)_2_ (**B**,**F**) and ANFO produced from gAN supplemented with 7 wt. % Ba(ClO_4_)_2_ using naphtha (**C**,**G**) and liquid paraffin (**D**,**H**) as fuel. Magnifications of 500× (**A**–**D**) and 5000× (**E**–**H**) for comparison.

**Figure A7:**
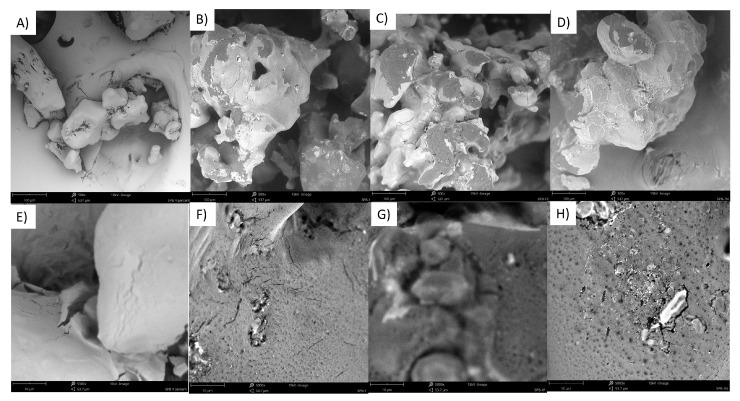
Compilation of SEM images for raw SPB (**A**,**E**), gAN supplemented with 1 wt. % SPB (**B**,**F**) and ANFO produced from gAN supplemented with 1 wt. % SPB using naphtha (**C**,**G**) and liquid paraffin (**D**,**H**) as fuel. Magnifications of 500× (**A**–**D**) and 5000× (**E**–**H**) for comparison.

**Figure A8:**
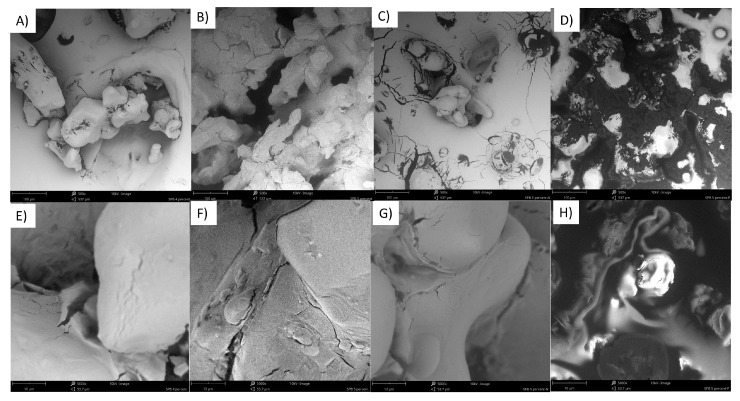
Compilation of SEM images for raw SPB (**A**,**E**), gAN supplemented with 5 wt. % SPB (**B**,**F**) and ANFO produced from gAN supplemented with 5 wt. % SPB using naphtha (**C**,**G**) and liquid paraffin (**D**,**H**) as fuel. Magnifications of 500× (**A**–**D**) and 5000× (**E**–**H**) for comparison.

**Figure A9:**
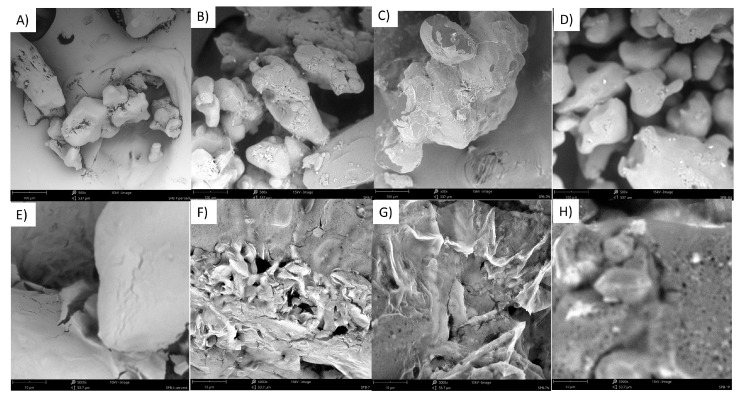
Compilation of SEM images for raw SPB (**A**,**E**), gAN supplemented with 7 wt. % SPB (**B**,**F**) and ANFO produced from gAN supplemented with 7 wt. % SPB using naphtha (**C**,**G**) and liquid paraffin (**D**,**H**) as fuel. Magnifications of 500× (**A**–**D**) and 5000× (**E**–**H**) for comparison.

**Figure A10:**
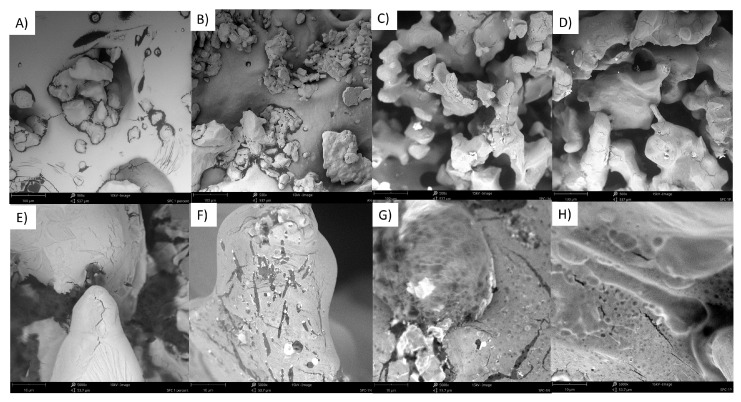
Compilation of SEM images for raw SPC (**A**,**E**), gAN supplemented with 1 wt. % SPC (**B**,**F**) and ANFO produced from gAN supplemented with 1 wt. % SPC using naphtha (**C**,**G**) and liquid paraffin (**D**,**H**) as fuel. Magnifications of 500× (**A**–**D**) and 5000× (**E**–**H**) for comparison.

**Figure A11:**
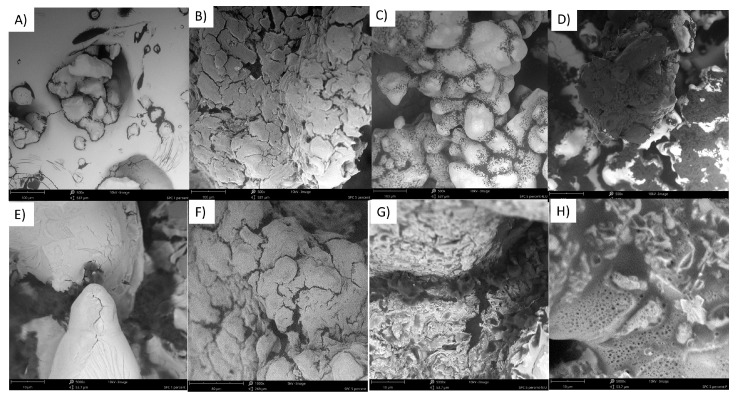
Compilation of SEM images for raw SPC (**A**,**E**), gAN supplemented with 5 wt. % SPC (**B**,**F**) and ANFO produced from gAN supplemented with 5 wt. % SPC using naphtha (**C**,**G**) and liquid paraffin (**D**,**H**) as fuel. Magnifications of 500× (**A**–**D**) and 5000× (**E**–**H**) for comparison.

**Figure A12:**
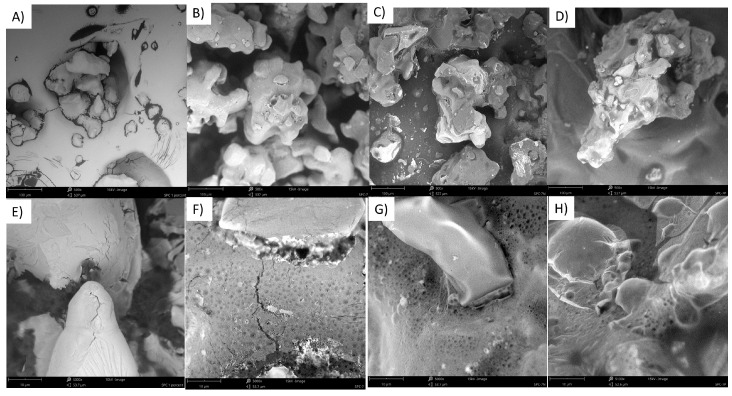
Compilation of SEM images for raw SPC (**A**,**E**), gAN supplemented with 7 wt. % SPC (**B**,**F**) and ANFO produced from gAN supplemented with 7 wt. % SPC using naphtha (**C**,**G**) and liquid paraffin (**D**,**H**) as fuel. Magnifications of 500× (**A**–**D**) and 5000× (**E**–**H**) for comparison.

Heating ANFO (10 K/min), produced using liquid paraffin, showed a change in the ignition temperature as a function of the utilised additive, in the following series (arranged from the lowest ignition temperature to the highest): SPB, SPC, Ba(ClO_4_)_2_, and Mg(ClO_4_)_2_. In the case of naphtha, the trend is as follows: Mg(ClO_4_)_2_, SPC, SPB, and Ba(ClO_4_)_2_. A sample recorded thermogram is presented in Figure 5 and the overall results of this investigation are summarised in Table 1.

The lower decomposition temperatures observed for samples supplemented with SPB and SPC are likely related to the low decomposition temperatures of these compounds. The impact of these additives is more pronounced in the case of ANFO produced using liquid paraffin, due to the higher viscosity of this fuel, which translates into more facile entrapment of gaseous SPC/SPB decomposition products inside this phase. Gas bubbles generated during this process might work as hot-spots, in a manner similar to bubbles generated during the chemical sensitization of explosives [14]. In the case of naphtha-based ANFO, the trend is similar, except for ANFO supplemented with Mg(ClO_4_)_2_, which shows a markedly lower decomposition temperature.

We have considered to what extent will the perchlorates yield highly explosive ammonium perchlorate upon reaction with the gAN fraction, as engineered in our previous works on emulsion explosives. This may be the case; however, the sensitization is observed only for ANFO supplemented with magnesium perchlorate rather than for both perchlorates. This indicates that the sensitization effect is specific to magnesium perchlorate rather than to any amount of precipitated ammonium perchlorate. This feature may be explained by the fact that while magnesium perchlorate exhibits moderate solubility in a variety of organic solvents, including dimethyl sulfoxide, diethyl ether, dichloromethane, and acetonitrile, barium perchlorate is noticeably less soluble in organic solvents [15,16,17]. The partial dissolution of magnesium perchlorate in naphtha yields a system that contains a highly reactive oxidising agent and a fuel in a single phase, facilitating a reaction between the two components. Even partial oxidation of naphtha in this system may generate enough heat to ignite the bulk of the ANFO, resulting in the observed low decomposition temperature.

The pseudo-activation energy values for the decomposition of ANFO samples supplemented with 7 wt. % (Table 2) were determined by the Kissinger and Ozawa methods. In all cases, the values determined with the two methods are in good agreement.

The activation energy for the decomposition of ANFO supplemented with Ba(ClO_4_)_2_ is on the order of 64–69 kJ/mol. Conversely, the literature reports activation energies on the order of 276–326 kJ/mol for the pure additive [18]. This discrepancy may be attributed to the formation of even trace amounts of ammonium perchlorate in the reaction between barium perchlorate and ammonium nitrate, as ammonium perchlorate has been reported to exhibit activation energy values for its decomposition reaction in the range of 60–90 kJ/mol [19,20]. This is well in line with the values calculated for perchlorate-supplemented ANFO. The observed differences between ANFO, produced using either Ba(ClO_4_)_2_ or Mg(ClO_4_)_2_, are expected to stem from differences in the maximum perchlorate anion concentrations achieved in the fuel phases in contact with gAN and, therefore, the amount of ammonium perchlorate precipitated within the samples.

In the case of the SPB-supplemented ANFO, the calculated E_A_ of 66–72 kJ/mol is significantly lower than the activation energy reported for the decomposition of pure SPB to the respective metaborate, which is on the order of 150–300 kJ/mol [21]. It should be noted here that it is impossible for the experimentally observed pseudo-activation energy to be related to the first step of SPB dehydration, despite it being reported to exhibit an E_A_ of 76 kJ/mol, due to the fact that this process achieves complete conversion below 100 °C, even at high heating rates [21]. Consequently, this discrepancy may be attributed to the interactions with AN, possibly with the formation of ammonium perborate, which would be expected to exhibit a notably lower activation energy of decomposition.

For samples supplemented with SPC, the pseudo-activation energy values were in the range of 81–85 kJ/mol. This is well in line with the values reported in the literature for the decomposition of SPC, which are on the order of 90–100 kJ/mol [22,23]. This similarity is indicative of the lack of any significant interactions between gAN or the utilised fuels and SPC, contrary to the above, where interactions or chemical reactions of the sample components led to significant discrepancies between the expected and observed E_A_ values.

In the case of samples that utilised naphtha as a fuel, the low boiling point (<90 °C) resulted in the evaporation of fuel from the system during the measurement. This effect was particularly pronounced at low heating rates but was significant even at a heating rate of 20 K/min. The uncertainty introduced by this process was found to be high enough to make any attempt at determining the pseudo-activation energy unreliable.

In comparison with the activation energies reported in the literature for the decomposition of ANFO (both pure and additive-supplemented), the pseudo-activation energy values determined via our experiments are noticeably lower. This is indicative of the viability of the selected additives as promoters of the ANFO decomposition. Among the investigated additives, SPB appears to be the most promising, as it offers the lowest effective pseudo-activation energy for ANFO decomposition, is a chlorine-free agent, and is often considered to be a “green” oxidising agent [24,25].

**Table 2:** Comparison of the pseudo-activation energies of decomposition for the investigated ANFO supplemented with 7 wt. % of the selected additives, determined via the Kissinger [26,27] and Ozawa [28] methods. Activation energy values reported in the literature for pure and additive-supplemented ANFO are included for further comparison.

Additive	Fuel	E_A_ ^1^ (Kissinger) [kJ/mol]	E_A_ ^1^ (Ozawa) [kJ/mol]
Ba(ClO_4_)_2_	paraffin	80.41	85.05
Mg(ClO_4_)_2_	paraffin	78.17	82.89
SPB	paraffin	66.69	71.92
SPC	paraffin	81.21	85.69
**Additive**	**Fuel**	**E_A_ [kJ/mol]**	**Source**
7% NaCl	-	147.6	[29]
5% BaCl_2_	-	145.4	[29]
5% NaF	-	162.5	[29]
-	5% Mineral oil	147	[30]
-	10% Dodecane	164	[30]
-	10% Mesitylene	165	[30]
-	4% Charcoal	166.7	[29]
-	8.6% Charcoal	122.6	[29]

^1^ E_A_—pseudo-activation energy of decomposition.

### 2.4. Impact and Friction Sensitivity Parameters

The friction sensitivity of ANFO produced using unmodified gAN was >360 N, regardless of whether liquid paraffin or naphtha was used as the organic fuel. The impact sensitivities of the two types of ANFO are 35 J and 40 J, respectively, for liquid paraffin and naphtha. The introduction of the selected additives has an expected impact in increasing the sensitivity of the resultant ANFO, regarding impact and friction (Table 3).

The inclusion of perchlorates results in a maximum observed impact sensitivity at 5 wt. % when using naphtha as fuel, whereas similar sensitivity is achieved only at a 7 wt. % content of the additive for ANFO produced using liquid paraffin. This indicates that even though rapid evacuation of the dispersing agent (acetone) was conducted during supplementation, recrystallisation of perchlorates is taking place, being more pronounced in the case of naphtha-based ANFO. Interestingly, for SPB and SPC, a 1 wt. % of the additive results in decreasing the sensitivity of ANFO to impact, but increasing the additive content brings about an increase in sample sensitivity, even if not as pronounced as in the case of the investigated perchlorates. It should also be noted that, due to the fact that the additives have been selected to increase the shock sensitivity of ANFO, the standardised methodology of investigating their impact sensitivity may not be entirely reliable. This is due to the fact that the impact of the Fallhammer may induce the adiabatic compression of air, particularly for large impact energies, effectively generating a weak shock wave, as has been pointed out in the literature [31]. The occurrence of such a shock wave may interfere with the actual result of the impact sensitivity test.

In the case of friction sensitivity, even a significant amount of the perchlorate additives does not necessarily result in high sensitivity. This is, however, highly dependent on the utilised additive and organic fuel, indicating the non-straightforward behavior of the additives in the ANFO samples. The difference between the observed trends in the impact and friction sensitivity of perchlorate-supplemented samples may stem from the fact that Ba(ClO_4_)_2_ and Mg(ClO_4_)_2_ crystallise in the hexagonal and monoclinic systems, respectively [32,33]. Such crystal geometry is more prone to tumbling, potentially making those substances act as “lubricants” in the friction sensitivity tests. The significant sensitization of naphtha-based ANFO supplemented with 7 wt. % Mg(ClO_4_)_2_ to friction may stem from the partial dissolution of Mg(ClO_4_)_2_ in the organic fuel, as mentioned in the discussion of the impact of the additives on ANFO decomposition temperatures.

In the case of SPB, the additive only has a limited influence on the friction sensitivity of ANFO, as even samples containing 7 wt. % SPB exhibit relatively minor sensitivity to friction. In turn, however, the addition of SPC, particularly in the case of naphtha-based ANFO samples, has a significant adverse influence on their friction sensitivity. This likely stems from the relatively low decomposition temperature of SPC (the decomposition onset is observed at approx. 100 °C) [34]. Friction-induced local overheating may promote the decomposition of SPC, which causes oxidation of the organic fuel (particularly, the more reactive naphtha).

## 3. Materials and Methods

### 3.1. Chemicals

Ammonium nitrate (AN) porous prill (with >99.4% purity, UltrAN 70, bulk density of 670–720 kg/m^3^, and prill diameters in the range of 1.0–2.0 mm) produced by Yara (Szczecin, Poland). Naphtha (technical grade, light, boiling point <90 °C) was obtained from Radchem (Grudziadz, Poland) and liquid paraffin (technical grade) was obtained from Chempur (Piekary Slaskie, Poland). Magnesium perchlorate (reagent grade) and barium perchlorate (>97% purity) were obtained from Sigma-Aldrich (St. Louis, MO, USA). Sodium carbonate peroxyhydrate (Na_2_CO3·1.5H_2_O_2_, **SPC**) and sodium perborate tetrahydrate (>97% purity, **SPB**) were obtained from Alfa Aesar (Kandel, Germany). Acetone (technical grade) was obtained from POCH S.A. (Gliwice, Poland).

### 3.2. Sample Preparation

AN was ground to achieve a powder-like grain size distribution, followed by sieving to separate grain fractions larger than 500 μm. The addition of a ground AN fraction to the porous AN prill fraction was reported to improve the detonation properties of ANFO produced using the two AN fractions [7]. In our case, grinding also facilitated the introduction of the additives selected for investigation into the bulk AN.

The selected additives, i.e., Mg(ClO_4_)_2_, Ba(ClO_4_)_2_, SPB, and SPC were dispersed in 20 cm^3^, 30 cm^3^, or 35 cm^3^ volumes, depending on the concentration of the additive, and were introduced into the ground AN (gAN), at a ratio of 0.2 g, 1 g, or 1.4 g of the additive dispersion per 20 g of AN, yielding samples containing 1 wt. %, 5 wt. %, and 7 wt. % of the additive, respectively. The samples were dried at 45–50 °C for 72 h, in order to evacuate acetone and any moisture absorbed by the gAN during processing. Subsequently, the additive-modified gAN was thoroughly mixed with either liquid paraffin or naphtha, to produce samples for testing friction and impact sensitivity, as well as their thermal characteristics, as a function of the type and amount of the utilised additive.

### 3.3. Material Characterisation

Friction and impact sensitivity were performed according to relevant international standards, using the Peters’ friction apparatus [35] and BAM Fallhammer apparatus [36], respectively.

Thermogravimetric (DTA-TG) measurements were conducted using a thermogravimetric analyzer, MOM Q1500, with a Paulik–Paulik–Erdey system. Samples of unmodified ANFO, as well as ANFO supplemented with varied amounts (1–7 wt. %) of the selected additive were placed in alumina crucibles and then placed in the DTA-TG oven and heated at 65–70 °C for 16 h. This was done in order to evaluate the potential impact of the utilised additives on the thermal stability of ANFO. The temperature set point (65–70 °C) was chosen so as to simultaneously maximize the rate of any ongoing decomposition processes and avoid inducing the phase transition of ammonium nitrate from α-rhombic to tetragonal, which occurs at approx. 84 °C [37].

The decomposition temperatures of the produced ANFO samples were determined using an Automatic Explosion Temperature 402 Tester (OZM Research, Bliznovice, Czech Republic). The decomposition temperature measurement was conducted five-fold (one “shot”, consisting of five separate samples) for each sample, and the final result is presented as an average. Samples of 50 ± 1 mg were used to determine the ignition/explosion temperatures. The measurement was carried out in the range of 100–400 °C. Heating rates of 5 K/min, 10 K/min, 15 K/min, and 20 K/min were employed to determine the pseudo-activation energy of decompositions for the investigated samples via the Kissinger [26,27] and Ozawa [28] methods.

The morphology of the components, additive-supplemented gAN, and respective ANFO were investigated using a Phenom ProX (Waltham, MA, USA) scanning electron microscope (SEM). Regarding the SEM operation parameters, the working distance was 10 mm, the acceleration voltage of the incident electron was 10 kV, and the current intensity of the incident electronic beam was about 95 μA.

## 4. Conclusions

The SEM investigation of the samples revealed that the introduction of the additives with the use of acetone as a carrier medium results in significantly increasing the surface porosity of gAN. Depending on the solubility of the additive, its introduction initially led to either a decrease or increase in gAN particle porosity. However, upon the introduction of the fuel, the porosity of the gAN was restored to an extent dependent on the solubility of the additive and the amount it was introduced in, with higher additive contents translating to less porous gAN particles in the final ANFO.

The investigation of the pseudo-activation energy for ANFO samples supplemented with the selected additives reveal E_A_ values significantly lower than those reported in the literature for additive-free ANFO and ANFO modified with NaF, NaCl, or BaCl_2_. This is indicative of the ability of the selected additives to promote the decomposition of ANFO, and based on the obtained results, initial hypotheses on the interactions between these additives with ANFO have been formulated for future experimental verification. Consequently, the four additives can be seen as viable in terms of improving the shock wave sensitivity of ANFO and should not be adversely affected by the use of different fuels.

An important discovery is that of the particularly low pseudo-activation energy for ANFO supplemented with SPB (E_A_ values of 66–72 kJ/mol), which is notably lower than for the other investigated additives, making SPB a promising additive to ANFO. Moreover, SPB is currently widely used as a “green”, chlorine-free, and inexpensive oxidising agent, which is relevant in terms of the economy of producing and utilising such modified ANFO formulations on an industrial scale.

The four investigated additives were found to modify the mechanical sensitivity of ANFO to some extent, but the magnitude of sensitization is within acceptable bounds, enabling the preparation of charges for follow-up testing in proving ground conditions, which will allow elucidating the impacts of these additives on the energetic parameters of supplemented ANFO.

In this work, ANFO was produced using two extreme cases of liquid organic fuels, i.e., naphtha and liquid paraffin, which are, respectively, low-boiling (<90 °C) and relatively high-boiling (>300 °C), in comparison with the traditional fuel oils (exhibiting boiling points in the range of 200–300 °C) used for producing ANFO. The use of naphtha as a fuel, due to its low boiling point, came with a set of challenges, as the evaporation and boiling-off of the fuel interfered with the results of thermochemical experiments. In practical conditions, the use of naphtha is not expected to be as problematic, but will likely significantly accelerate the aging of ANFO.

Modification of the shock wave sensitivity of ANFO using the investigated additives requires careful optimisation, both in terms of economic factors and physicochemical interactions between them and the other components of the produced ANFO. The issue of partial solubility of magnesium perchlorate in low-boiling organic fuel is of importance, as is the potential for the recrystallisation of the additives. The former may be easily remedied by the replacement of part of the gAN fraction with magnesium nitrate, which would be expected to saturate the naphtha or other low-boiling fuel with Mg^2+^ cations, drastically reducing the solubility of Mg(ClO_4_)_2_ in that fuel. The latter calls for developing a more technologically viable method of introducing the additives into the gAN fraction.

The observed changes in the impact sensitivity of the modified ANFO samples as a function of the amount and type of additive deviate from what would be expected based on general theory. These deviations may indicate a marked increase in shock wave sensitivity, as the samples may be initiated by the adiabatic compression of air in the sample holder upon the impact of the Fallhammer. This hypothesis, however, needs to be verified in further research, by conducting a gap test. It would also be beneficial to investigate the impact sensitivity of the ANFO samples via a method in which the samples are unconfined, e.g., in the ball drop test [31], and corroborate those results with the impact sensitivity obtained via the standard BAM Fallhammer test conducted in this work.

## Figures and Tables

**Table 1:** Ignition/explosion temperature (I/ET) values observed for the investigated ANFO samples supplemented by 7 wt. % of the investigated additives.

Shot No.	Sample	Fuel	Heating Rate	I/ET [°C]
1	ANFO	naphtha	10 °C/min	223
2	ANFO	paraffin	10 °C/min	276
3	Ba(ClO_4_)_2_	naphtha	10 °C/min	250
4	Ba(ClO_4_)_2_	paraffin	10 °C/min	268
5	Mg(ClO_4_)_2_	naphtha	10 °C/min	164
6	Mg(ClO_4_)_2_	paraffin	10 °C/min	269
7	SPB	naphtha	10 °C/min	247
8	SPB	paraffin	10 °C/min	254
9	SPC	naphtha	10 °C/min	232
10	SPC	paraffin	10 °C/min	249

**Table 3:** Impact of introducing the selected additives into g-AN on the impact and friction sensitivity parameters of ANFO.

Impact sensitivity [J] (fuel: naphtha)				
	**Amount**	**1 wt. %**	**5 wt. %**	**7 wt. %**
**Additive**				
Mg(ClO_4_)_2_		40	4	10
Ba(ClO_4_)_2_		40	4	10
SPB ^1^		>50	35	20
SPC ^2^		>50	7.5	-
**Impact sensitivity [J] (fuel: liquid paraffin)**				
	**Amount**	**1 wt. %**	**5 wt. %**	**7 wt. %**
**Additive**				
Mg(ClO_4_)_2_		25	15	3
Ba(ClO_4_)_2_		20	15	3
SPB ^1^		>50	>50	7.5
SPC ^2^		>50	35	-
**Friction sensitivity [N] (fuel: naphtha)**				
	**Amount**	**1 wt. %**	**5 wt. %**	**7 wt. %**
**Additive**				
Mg(ClO_4_)_2_		288	288	120
Ba(ClO_4_)_2_		288	324	360
SPB ^1^		>360	252	216
SPC ^2^		>360	112	-
**Friction sensitivity [N] (fuel: liquid paraffin)**				
	**Amount**	**1 wt. %**	**5 wt. %**	**7 wt. %**
**Additive**				
Mg(ClO_4_)_2_		252	144	216
Ba(ClO_4_)_2_		180	324	216
SPB ^1^		>360	214	252
SPC ^2^		324	252	-

^1^ SPB—sodium perborate tetrahydrate. ^2^ SPC—sodium carbonate peroxyhydrate (Na_2_CO3·1.5H_2_O_2_).

## Data Availability

Data are available from the authors on request.
